# Supplementary material for: HIVprotI: an integrated web based platform for prediction and design of HIV proteins inhibitors
Source: J Cheminform. 2018 Mar 9;10:12. doi: 10.1186/s13321-018-0266-y (PMC5845081; doi:10.1186/s13321-018-0266-y)
Supplement: Supplementary file 2 — Additional file 2. Source code of HIVProtI web server. [file 13321_2018_266_MOESM2_ESM.zip › HIVprotI_Source-code/hivprotI/weka-3-6-6/doc/weka/attributeSelection/BestFirst.LinkedList2.html]

BestFirst.LinkedList2


---


|  |  |  |
| --- | --- | --- |
| **Overview** \| **Package** \| **Class** \| **Tree** \| **Deprecated** \| **Index** \| **Help** \| **Weka's home** | |  |
| **PREV CLASS**   **NEXT CLASS** | **FRAMES**    **NO FRAMES**     **All Classes** |  |
| SUMMARY: NESTED \| FIELD \| CONSTR \| METHOD | DETAIL: FIELD \| CONSTR \| METHOD |  |


---


## weka.attributeSelection Class BestFirst.LinkedList2

```
java.lang.Object
  weka.core.FastVector
      weka.attributeSelection.BestFirst.LinkedList2
```

**All Implemented Interfaces:**: java.io.Serializable, Copyable, RevisionHandler

**Enclosing class:**: BestFirst

---

``` public class BestFirst.LinkedList2 extends FastVector ```

Class for handling a linked list. Used in best first search.
Extends the Vector class.

**Author:**
:   Mark Hall (mhall@cs.waikato.ac.nz)

**See Also:**: Serialized Form

---

**Nested Class Summary**

**Nested classes/interfaces inherited from class weka.core.FastVector**

`FastVector.FastVectorEnumeration`


**Constructor Summary**

`BestFirst.LinkedList2(int sz)`


| **Method Summary** | |
| --- | --- |
| `void` | `addToList(java.lang.Object[] data, double mer)`             adds an element (Link) to the list. |
| `BestFirst.Link2` | `getLinkAt(int index)`             returns the element (Link) at a specific index from the list. |
| `java.lang.String` | `getRevision()`             Returns the revision string. |
| `void` | `removeLinkAt(int index)`             removes an element (Link) at a specific index from the list. |

**Methods inherited from class weka.core.FastVector**

`addElement, appendElements, capacity, contains, copy, copyElements, elementAt, elements, elements, firstElement, indexOf, insertElementAt, lastElement, removeAllElements, removeElementAt, setCapacity, setElementAt, size, swap, toArray, trimToSize`

**Methods inherited from class java.lang.Object**

`equals, getClass, hashCode, notify, notifyAll, toString, wait, wait, wait`

**Constructor Detail**

### BestFirst.LinkedList2

```
public BestFirst.LinkedList2(int sz)
```


**Method Detail**

### removeLinkAt

```
public void removeLinkAt(int index)
                  throws java.lang.Exception
```

:   removes an element (Link) at a specific index from the list.

    :   **Parameters:**: `index` - the index of the element to be removed. **Throws:**: `java.lang.Exception`

---


### getLinkAt

```
public BestFirst.Link2 getLinkAt(int index)
                          throws java.lang.Exception
```

:   returns the element (Link) at a specific index from the list.

    :   **Parameters:**: `index` - the index of the element to be returned. **Throws:**: `java.lang.Exception`

---


### addToList

```
public void addToList(java.lang.Object[] data,
                      double mer)
               throws java.lang.Exception
```

:   adds an element (Link) to the list.

    :   **Parameters:**: `data` - the attribute set specification: `mer` - the "merit" of this attribute set **Throws:**: `java.lang.Exception`

---


### getRevision

```
public java.lang.String getRevision()
```

:   Returns the revision string.

    :   **Specified by:**: `getRevision` in interface `RevisionHandler` **Overrides:**: `getRevision` in class `FastVector`
    :   **Returns:**: the revision


---


|  |  |  |
| --- | --- | --- |
| **Overview** \| **Package** \| **Class** \| **Tree** \| **Deprecated** \| **Index** \| **Help** \| **Weka's home** | |  |
| **PREV CLASS**   **NEXT CLASS** | **FRAMES**    **NO FRAMES**     **All Classes** |  |
| SUMMARY: NESTED \| FIELD \| CONSTR \| METHOD | DETAIL: FIELD \| CONSTR \| METHOD |  |


---
